# Efficacy and Safety of Daraxonrasib (RMC-6236) in Previously Treated Metastatic RAS-Mutated Pancreatic Ductal Adenocarcinoma: A Systematic Review

**DOI:** 10.3390/cancers18193112

**Published:** 2026-09-25

**Authors:** Natalia Gierulska, Nina Jankowska, Zuzanna Dąbrowska, Julia Piekarz, Magdalena Skórzewska

**Affiliations:** 1 Student Scientific Society, Department of Clinical Oncology and Chemotherapy, Medical University of Lublin, 20-059 Lublin, Poland; 61976@umlub.edu.pl (N.J.); 61934@umlub.edu.pl (Z.D.); 59745@umlub.edu.pl (J.P.); 2 Department of Clinical Oncology and Chemotherapy, Medical University of Lublin, 20-059 Lublin, Poland

**Keywords:** daraxonrasib, RMC-6236, RAS inhibitor, pancreatic ductal adenocarcinoma, KRAS, systematic review, targeted therapy

## Abstract

**Introduction**: Pancreatic ductal adenocarcinoma (PDAC) is associated with a poor prognosis, and *RAS* gene mutations are present in more than 90% of cases. For this reason, direct *RAS* inhibition is a promising, but thus far difficult to achieve, therapeutic strategy. Daraxonrasib (RMC-6236) is a first-in-class, oral, non-covalent, multiselective inhibitor of the active form of RAS. It forms an intracellular trimeric complex with cyclophilin A, blocking both mutant and wild-type variants of the *KRAS, NRAS,* and HRAS genes. The aim of this systematic review was to evaluate the efficacy and safety of daraxonrasib in previously treated patients with *RAS*-mutated PDAC. **Methods:** This review was conducted in accordance with the PRISMA 2020 guidelines. A systematic search was conducted in PubMed, Web of Science, and ClinicalTrials.gov for studies published between January 2020 and July 2026. Two studies met the inclusion criteria and were analyzed: the Phase 1–2 RMC-6236-001 (*n* = 168) and the Phase 3 RASolute 302 trial (*n* = 500), involving a total of 668 patients. The risk of bias was assessed using the Cochrane RoB 2.0 tool for the randomized trial and the Methodological Index for Non-Randomized Studies (MINORS) tool. **Results:** In the RASolute 302 trial, daraxonrasib out performed investigator-selected chemotherapy in a population of patients with the *RAS* G12 mutation, improving median overall survival (13.2 vs. 6.6 months; HR 0.40), progression-free survival (7.3 vs. 3.5 months; HR 0.45), and objective response rate (33.2% vs. 11.8%; all *p* < 0.001), and also delayed the onset of pain and deterioration in quality of life. These results were consistent with the earlier RMC-6236-001 study (objective response rate [ORR] 35%, median overall survival [OS] 13.1 months in patients with *RAS* G12 mutation treated in the second-line setting). Daraxonrasib exhibited a predictable toxicity profile, consisting mainly of Grade 1–2 dermatologic and gastrointestinal symptoms, with lower rates of hematologic toxicity and treatment discontinuation than with chemotherapy. **Conclusions:** Daraxonrasib demonstrates clinically relevant and statistically significant efficacy with an acceptable safety profile in previously treated patients with metastatic *RAS* G12-mutated PDAC, the population for which the strongest randomized evidence is currently available. These findings support its potential as a promising new treatment option in this setting. Further studies are needed regarding rare *RAS* gene variants and forms of the disease *with wild-type RAS*.

## Simple Summary

Pancreatic cancer is one of the most aggressive forms of cancer, and in the majority of cases involves a mutation in the *RAS* gene, a protein which, when activated, stimulates tumour growth. Inhibiting this protein has long been a challenge, and previous drugs were effective against only one rare type of mutation. In August 2026, a new oral drug called daraxonrasib was approved, as it is capable of blocking the active form of RAS across multiple mutation types, offering hope to a greater number of patients. This review brings together the available data from clinical trials on the efficacy and safety of daraxonrasib in patients whose pancreatic cancer has progressed following prior treatment. The results show that daraxonrasib prolonged survival, slowed disease progression and led to tumour shrinkage more frequently than chemotherapy, whilst causing mainly mild, manageable side effects. These results confirm that daraxonrasib is a promising new drug and also highlight areas where further research is still needed, particularly with regard to rarer types of mutations.

## 1. Introduction

Pancreatic ductal adenocarcinoma (PDAC) is one of the most common histological types of malignant pancreatic tumors and accounts for over 90% of all diagnosed cases [1]. PDAC is characterized by an aggressive clinical course and a poor prognosis. The 5-year survival rate in patients with PDAC is 11–13% [1,2]. According to global GLOBOCAN 2022 statistics, there were approximately 511,000 new cases and 467,000 deaths from this cancer. For this reason, pancreatic cancer currently ranks 12th in terms of incidence and 6th in terms of cancer-related deaths worldwide [1]. A particular challenge is the increasing incidence of early-onset forms in an increasingly younger population. This form shows a clear correlation with non-modifiable and familial risk factors, including germline mutations in the *BRCA2* and *PALB2* genes and genetic syndromes, as well as with lifestyle and chronic pancreatitis [2]. In addition to classic PDAC, rarer, aggressive histologic variants, such as pancreatic adenocarcinoma with squamous differentiation (PASC), pose a serious clinical challenge. It is characterized by a significantly poorer prognosis and a higher rate of lymphatic and nerve invasion compared to classic PDAC [3]. Pancreatic cancer is a tumor that lacks specific clinical symptoms in its early stages, resulting in an extremely high mortality rate. Additionally, traditional diagnostic methods such as high-resolution computed tomography (CECT) and endoscopic ultrasound (EUS) have reduced sensitivity in detecting microscopic lesions smaller than 2 cm [4]. Furthermore, in patients with the Lewis-negative phenotype, the CA 19-9 marker unfortunately has low sensitivity, as they do not synthesize the antigen [2,5]. For patients with localized PDAC, surgical resection currently remains the only chance for long-term cure. According to current NCCN and ESMO clinical practice guidelines, neoadjuvant therapy (NAT) is established as the mandatory standard of care for borderline resectable and locally advanced PDAC to maximize R0 resection rates and eliminate micrometastatic disease. NAT is also increasingly recommended for upfront resectable PDAC in the presence of high-risk clinical features, reflecting a shift towards early systemic intervention in the treatment of pancreatic cancer [6,7].

Due to the dynamic nature of the tumor and the frequent occurrence of chemoresistance, the concept of adaptive therapy has become an extremely important element of preoperative treatment. Studies show that approximately 20% of patients with localized PDAC require rotation of the first-line regimen due to radiological progression, lack of CA 19-9 response, or toxicity. This allows for resection in 42% of patients in this group and achieves an OS comparable to that of patients responding to first-line therapy [8].

In the generalized and metastatic stages, treatment is based on aggressive multi-drug regimens, including FOLFIRINOX and gemcitabine in combination with nab-paclitaxel [9,10]. Recent findings from the NAPOLI 3 study have also confirmed the clinical superiority of the new NALIRIFOX regimen as the first-line standard of care for advanced PDAC [9]. To optimize systemic therapy, synergistic methods are being explored, including physical ablation techniques such as focused ultrasound (FUS/HIFU) [11]. The challenges in treating PDAC stem largely from the unique characteristics of the tumor microenvironment (TME), which acts as a strong physical and immunological barrier [12,13,14]. At the same time, PDAC is characterized by an extremely immunosuppressive profile, dominated by regulatory T cells (Tregs), myeloid-derived suppressor cells (MDSCs), and tumor-associated macrophages (TAMs), which effectively block the activation of CD8+ cytotoxic T cells [12]. The inflammatory axis, regulated by COX-2, NF-κB, and the Sp1 transcription factor, plays a significant role in promoting immunosuppression and invasiveness. Their overexpression in tumor tissue correlates with significantly poorer overall survival in patients [13].

To date, regulatory successes in the field of direct RAS protein inhibition were limited exclusively to covalent inhibitors targeting the inactive state of the protein (RAS-OFF), which precisely block only the *KRAS* G12C mutation. This group includes sotorasib (AMG 510) and adagrasib (MRTX849), which have received FDA approval for use as monotherapy in NSCLC and in combination therapy for colorectal cancer (CRC) [14,15]. Although their approval marked a historic breakthrough, they have strict therapeutic limitations. They are completely inactive against other oncogenic variants due to the absence of a reactive cysteine at codon 12 [14]. In pancreatic cancer, the *G12C* mutation occurs in only 1–3% of patients, whereas the predominant variants are G12D (44%), G12V (34%), and G12R (20%), which do not respond to currently approved RAS-OFF drugs [15]. Furthermore, for other aggressive cancers, such as *NRAS*-mutated melanoma, there were previously no FDA-approved targeted therapeutic options at all [13].

The situation changed on 26 August 2026, when the FDA approved daraxonrasib (RASONQUE) as a first-in-class non-covalent, multiselective inhibitor of the active state of RAS (RAS-ON) for patients with metastatic PDAC who have previously received at least one course of systemic therapy or who are not eligible for multiagent systemic therapy [16]. Daraxonrasib works by forming a tri-complex with cyclophilin A and simultaneously blocks *KRAS*, *NRAS*, and *HRAS* mutations [14,17].

Despite the recent publication of data from clinical trials on daraxonrasib, the scientific literature still lacks a comprehensive, critically assessed review of its clinical efficacy and safety based on the available studies. This systematic review collects and evaluates the full range of clinical evidence concerning daraxonrasib in the treatment of previously treated metastatic PDAC. Based on an analysis of the data, it provides an assessment of the therapeutic efficacy, safety profile and clinical potential of this drug compared with conventional cytotoxic treatment regimens and other strategies.

## 2. Materials and Methods

### 2.1. Study Designs

This systematic review was conducted in accordance with the Preferred Reporting Items for Systematic Reviews and Meta-Analyses (PRISMA) 2020 guidelines. The aim of the review was to comprehensively assess the efficacy and safety of daraxonrasib (RMC-6236) in the treatment of advanced or metastatic pancreatic ductal adenocarcinoma (PDAC). The analysis included different types of studies: randomized controlled trials (RCTs) and single-arm studies, to provide a broad perspective on available evidence. Because daraxonrasib has only recently been introduced into clinical trials, the number of available studies meeting the inclusion criteria was limited.

The review protocol was registered in the International Prospective Register of Systematic Reviews (PROSPERO) under registration number CRD420261493511. The PRISMA checklist can be found in the Supplementary.

### 2.2. Eligibility Criteria and PICO Framework

The research focused on evaluating the efficacy and safety of the therapy. The PICO model was used to structure and focus the literature review.

- Population: The target population consists of adult patients suffering from previously treated PDAC, harboring *RAS* mutations or *RAS* wild-type status.
- Intervention: Interventions include the use of daraxonrasib monotherapy (oral administration at doses up to 300 mg once daily or standard phase 3 dosing of 300 mg daily).
- Comparison: A comparative analysis of daraxonrasib therapy versus investigator’s choice of standard chemotherapy was conducted. For single-arm studies, results were analysed without comparison with a control group.
- Outcome: The results analyzed in the review focused on evaluating the efficacy and safety of daraxonrasib therapy in patients with PDAC. Efficacy was primarily assessed based on objective response rate (ORR), progression-free survival (PFS), overall survival (OS), duration of response (DoR), and patient-reported outcomes (time to deterioration in pain and global health status/quality of life). The safety of the therapy was analyzed by assessing the incidence of treatment-related adverse events (TRAEs), including serious adverse events (≥Grade 3).

Studies published in English between 1 January 2020, and 31 July 2026, were included in the analysis. RCTs and single-arm studies were included if they contained data on the use of daraxonrasib in the patient population in question. Exclusion criteria included review publications, reports of preclinical studies (both in animal and cellular models), abstracts without complete data (e.g., conference abstracts or posters that did not provide full results), and papers that did not include information on the efficacy or safety of daraxonrasib treatment.

### 2.3. Search and Selection Process

A systematic search of PubMed, Web of Science, and ClinicalTrials.gov was performed to identify relevant studies published between 1 January 2020, and 31 July 2026. PubMed/MEDLINE, Web of Science Core Collection, and ClinicalTrials.gov were selected as the primary databases because they represent, respectively, the gold-standard biomedical literature index, a comprehensive multidisciplinary citation database with strong oncology conference-abstract coverage, and the authoritative registry of interventional clinical trials. The final search was conducted on 1 September 2026. Incorporating an additional search in Scopus over the same timeframe yielded no further eligible studies, confirming that the current literature base for RMC-6236 in PDAC is fully captured by the two included trials. To address potential coverage gaps relative to EMBASE, this additional search was performed in Scopus, which provides substantial indexing overlap with EMBASE-listed biomedical journals; the search was conducted using an equivalent search string over the identical timeframe. As the Scopus search did not identify any additional unique records beyond those already captured in the primary three-database search, these results were not incorporated into the PRISMA flow diagram (Figure 1), which reflects the original search across PubMed, Web of Science, and ClinicalTrials.gov. The search strategies combined terms related to the target agent (“Daraxonrasib” OR “RMC-6236” OR “RAS inhibitor” OR “pan-RAS inhibitor”) with terms defining the disease entity (“pancreatic ductal adenocarcinoma” OR “pancreatic cancer” OR “PDAC”) using Boolean operators (AND/OR), field tags, and database-specific syntax. Broad search terms were intentionally utilized to ensure maximum sensitivity and capture all relevant early-phase clinical and translational reports, accounting for the initial yield of 1145 records. The full, verbatim search strings executed for each database—including Boolean operators, field tags (e.g., [Title/Abstract]), applied limits, and date restrictions—are detailed in Supplementary Materials (Table S1). To ensure the completeness of the review, a manual search of the results and an evaluation of the bibliographies of the selected articles was also conducted. Two researchers (J.P. and N.G.) independently searched the databases and critically appraised the selected articles. Any discrepancies were resolved by verification by two other authors (N.J. and Z.D.)

A systematic database search identified 1145 records, of which 730 records were removed before screening (680 duplicates, 40 marked as ineligible, and 10 removed for other reasons). Of the remaining 415 screened records, 330 were excluded during title/abstract screening. A total of 85 reports were sought for retrieval, of which 48 could not be retrieved. Most of these 48 unobtainable papers were conference abstracts, indexed separately in the Web of Science database, for which neither the full text nor a peer-reviewed publication was available at the time of the search. Additionally, some reports corresponded to entries in the ClinicalTrials.gov registry for which no related peer-reviewed publications or published results were available at the time of the search. Among the 37 reports assessed for full-text eligibility, 35 were excluded due to ineligible study design (*n* = 15), incorrect population (*n* = 12), or wrong intervention (*n* = 8). Ultimately, 2 articles met the inclusion criteria and were incorporated into the primary analysis. Following a quality assessment, all 2 studies were deemed to be of high methodological quality. The comprehensive data selection and identification process is illustrated in Figure 1.

### 2.4. Data Extraction and Synthesis

The review included one RCT and one single-arm study involving a total of 668 participants diagnosed with PDAC and treated with daraxonrasib or standard chemotherapy across trials (168 patients from the phase 1–2 trial and 500 patients from the phase 3 trial). Data extraction was performed independently by two reviewers using a standardized data extraction form. Extracted data included study characteristics (author, year, study design), patient demographics (total number of patients, age, gender), intervention details (daraxonrasib dosage, regimen), and outcome measures related to efficacy (ORR, PFS, OS, DoR, Complete Response (CR), Partial Response (PR), Stable Disease (SD), Progressive Disease (PD), Disease Control Rate (DCR)) and safety (incidence and severity of TRAEs). Any discrepancies were resolved through discussion or consultation with a third reviewer.

Overall, the synthesis included one RCT and one single-arm study, involving a total of 668 participants diagnosed with PDAC and treated across trials. A quantitative meta-analysis was intentionally not performed due to substantial methodological and clinical heterogeneity between the included studies, specifically comparing a single-arm Phase I/II dose-escalation trial (RMC-6236-001) with a randomized Phase III trial (RASolute 302). Pooling quantitative estimates across such disparate study designs would yield statistically invalid and biased outcomes; thus, a structured narrative synthesis was conducted.

### 2.5. Assessment of Risk of Bias in the Included Studies

The included clinical studies comprised both a randomized controlled trial (RASolute 302) and a single-arm interventional study (RMC-6236-001). Methodological quality and risk of bias were independently evaluated by two reviewers, with any discrepancies resolved through consensus or adjudication by a third reviewer.

For the randomized trial (RASolute 302), risk of bias was assessed using the Cochrane Risk of Bias 2 (RoB 2) tool across five standard domains: bias arising from the randomization process, bias due to deviations from intended interventions, bias due to missing outcome data, bias in measurement of the outcome, and bias in selection of the reported result. To rigorously account for the trial’s open-label design, outcome-specific risk-of-bias assessments were performed by stratifying endpoints into objective efficacy outcomes, such as OS and PFS evaluated by BICR-PFS, and subjective efficacy and safety outcomes, including TEAEs, Patient-Reported Outcomes (PROs), and investigator-assessed PFS. Domain-level judgments for the randomized trial are summarized in Figure 2, with detailed itemized rationales presented in Supplementary Table S2.

For the single-arm interventional trial (RMC-6236-001), methodological quality and risk of bias were formally evaluated using the Methodological Index for Non-Randomized Studies (MINORS), a validated instrument specifically designed for non-randomized interventional studies. The MINORS tool assesses eight specific domains for single-arm studies: (1) clearly stated aim, (2) inclusion of consecutive patients, (3) prospective collection of data, (4) endpoints appropriate to the aim, (5) unbiased assessment of study endpoints, (6) follow-up period appropriate to study aim, (7) loss to follow-up <5%, and (8) prospective calculation of study size. Each domain is scored as 0 (not reported), 1 (reported but inadequate), or 2 (reported and adequate), yielding a maximum score of 16. The RMC-6236-001 trial achieved a total MINORS score of 14 out of 16, indicating high overall methodological quality and low risk of major bias, with minor points deducted due to the open-label design (lack of blinded outcome assessment) and exploratory sample size determination for specific cohorts. Item-level evaluation, domain scores, and supporting rationale are provided in Supplementary Materials (Table S3).

This tailored approach to risk of bias assessment, specific to each study design, ensured a comprehensive evaluation of study quality and enhanced the overall reliability of the review findings.

## 3. Results

### 3.1. Mechanism of Action of Daraxonrasib

Daraxonrasib acts by forming a ternary complex that overcomes structural barriers and acts directly on the RAS protein [18,19]. The mechanism of action of this drug at the subcellular level occurs in several stages. Initially, after penetrating into the cancer cell, daraxonrasib binds to cyclophilin A (CypA) due to its high affinity. CypA is a chaperone protein found in the cell cytosol that, together with daraxonrasib, forms a stable daraxonrasib-CypA binary complex. In this process, CypA functions both as an intracellular receptor for the drug and as its cofactor. The resulting daraxonrasib-CypA complex possesses a unique structural surface that exhibits high affinity for the GTP-bound RAS protein (RAS(ON)). The binary complex conforms to the structure of RAS(ON) and forms a stable, non-covalent daraxonrasib-CypA-RAS(ON) tri-complex. This results in the formation of a steric block. The presence of the macromolecular CypA in the immediate vicinity of the Switch-I and Switch-II regions of the RAS molecule creates a physical spatial barrier. This barrier substantially impairs the binding and activation of key effector proteins in the pathway. In particular, it prevents effective interaction with RAF family kinases, such as CRAF or BRAF. As a result of the RAS oncoprotein remaining bound to GTP, its ability to stimulate the MAPK pathway is significantly suppressed. This causes a significant reduction in both proliferative and survival signals in the cancer cell [19]. To further clarify this multistep biochemical mechanism and its downstream functional consequences, Figure 3 provides a schematic illustration of ternary complex formation and the resulting suppression of RAS-MAPK signaling.

Daraxonrasib exhibits affinity for a broad spectrum of active RAS protein variants. It demonstrates activity against both the most common mutations, such as *KRAS* G12D, G12V, and G12R, as well as mutations at codons 13 and 61, and the wild-type forms of all three isoforms: *KRAS*, *NRAS*, and *HRAS*. As a result, daraxonrasib provides substantial and sustained suppression of oncogenic signaling. Additionally, it attenuates cancer cell escape through the activation of alternative RAS isoforms, which is a recognized contributor to resistance in previous therapies [18,19].

### 3.2. Pharmacokinetics and Translational Modeling of Pharmacokinetic-Pharmacodynamic Relationships

Daraxonrasib exhibits favorable pharmacokinetic (PK) properties that enable effective and sustained blockade of the RAS-MAPK signaling pathway in patients. Following oral administration, the drug is rapidly absorbed from the gastrointestinal tract, reaching maximum serum concentration (Tmax) within 1.6 to 3.9 h. Over the dose range of 80 mg to 300 mg per day, drug exposure (AUC and Cmax) increases proportionally with the administered dose. The mean terminal elimination half-life (t1/2) is approximately 11.8 h; therefore, the dosing regimen for this drug is once daily. Following repeated daily administration, daraxonrasib reaches steady-state plasma concentrations as a result of moderate accumulation of the drug. Translational studies and mathematical modeling of pharmacokinetic-pharmacodynamic (PK/PD) relationships have shown that sustained regression of PDAC requires constant and profound inhibition of RAS-MAPK pathway activity at a level of at least 90%. Daraxonrasib achieves this high threshold through preferential accumulation in tumor tissue, which does not occur in plasma or normal tissues. This enables the local maintenance of therapeutic concentrations that exceed the IC50 values for oncogenic RAS variants [19].

### 3.3. Daraxonrasib in the RMC-6236-001 and RASolute 302 Clinical Trials

Data on the clinical efficacy of daraxonrasib in PDAC are derived primarily from two studies: the single-arm Phase 1–2 RMC-6236-001 study [19] and the randomized Phase 3 RASolute 302 study [18]. The characteristics of both studies are presented in Table 1.

The RMC-6236-001 study evaluated 168 patients with previously treated **RAS**-mutated PDAC who received at least one dose of daraxonrasib not exceeding 300 mg [19]. The median age was 65 years, and 68% of participants had an ECOG performance status of 1. In 42% of patients, daraxonrasib was administered as second-line therapy, while 58% received it as third-line or subsequent-line therapy. *RAS G12* mutations were present in 149 patients, or 89% of the study population, and the most commonly identified variants were *KRAS* G12D, G12V, and *G12R* [19].

Antitumor activity was evaluated across dose levels and treatment-line subgroups. Numerically, the highest response rates were observed among patients receiving daraxonrasib at 300 mg in the second-line setting; however, these subgroup comparisons were exploratory and were not designed for formal between-group inference [19]. Detailed treatment response results are presented in Table 2.

In the group of patients with *RAS G12* mutations receiving 300 mg of daraxonrasib as second-line therapy, the ORR was 35% (95% CI: 17–56), while disease control was achieved in 92% of patients. The median time to response was 2.6 months, and the median DoR was 8.2 months. The median PFS was 8.5 months, while the median OS was 13.1 months. The probability of remaining progression-free at 6 and 9 months was 78% and 46%, respectively, and the probability of survival at 9 months was 89% [19].

In a larger cohort of patients with *RAS G12, G13*, or *Q61* mutations treated with a 300 mg dose in the second-line setting, the ORR was 29% and the DCR was 95%. Responses were observed, among others, in patients with the *KRAS G12D, G12V, G12R*, and *Q61H* variants. The median DoR was 8.2 months, the median PFS was 8.1 months, and the median OS was 15.6 months. After 9 months, 38% of patients remained progression-free, and 79% were still alive [19].

Numerically lower response rates were observed among patients treated in the third- or later-line setting. The ORR was 21% in the *RAS G12* subgroup and 20% in the overall *RAS*-mutated population, with median DoR values of 3.5 and 3.3 months, respectively [19]. These results should not, however, be interpreted as evidence of greater daraxonrasib efficacy when administered in an earlier treatment line. RMC-6236-001 was a single-arm Phase 1–2 study, and comparisons between second-line and later-line cohorts were exploratory, unadjusted cross-subgroup comparisons rather than prespecified randomized comparisons. The relatively small subgroup sizes further limit the precision of these estimates. Differences in outcomes may therefore reflect imbalances in patient characteristics, including performance status, prior treatment burden, disease characteristics, or other measured and unmeasured clinical factors. Consequently, no causal inference regarding the effect of treatment line on daraxonrasib efficacy can be made from these data [19].

### 3.4. Results of the Phase 3 RASolute 302 Trial

The activity of daraxonrasib observed in the early-phase trial was subsequently validated in the RASolute 302 trial [18]. This international, open-label Phase 3 trial enrolled 500 patients with metastatic PDAC who had experienced disease progression after one prior line of treatment. Patients were randomly assigned to receive daraxonrasib at a dose of 300 mg once daily (*n* = 248) or investigator-selected chemotherapy (*n* = 252). In the control arm, gemcitabine with nab-paclitaxel, liposomal irinotecan with fluorouracil and leucovorin, modified FOLFIRINOX, or FOLFOX were used. The *RAS G12* mutation was detected in 91.8% of participants [18].

The dual primary endpoints were OS and PFS in the *RAS G12* population. PFS and treatment response were assessed by blinded, independent central review in accordance with RECIST 1.1 criteria. The key results of the study are presented in Table 3.

In the *RAS G12* population, treatment with daraxonrasib prolonged median OS from 6.6 months with chemotherapy to 13.2 months, corresponding to a 60% reduction in the hazard of death (HR, 0.40; 95% CI, 0.30–0.54; *p* < 0.001). A similar benefit was observed in the overall population, in which median OS was 13.2 months with daraxonrasib and 6.7 months with chemotherapy (HR, 0.40; 95% CI, 0.30–0.53; *p* < 0.001) [18]. The OS results were generally consistent across the prespecified clinical subgroups, although estimates for rare molecular subgroups were less precise because of the limited number of patients and events [18].

A significant benefit was also demonstrated for PFS. In the *RAS G12* population, median PFS was 7.3 months with daraxonrasib compared with 3.5 months with chemotherapy (HR for disease progression or death, 0.45; 95% CI, 0.34–0.59; *p* < 0.001). In the overall population, median PFS was 7.2 and 3.6 months, respectively (HR, 0.49; 95% CI, 0.38–0.64; *p* < 0.001) [18]. At 6 months, 58.7% of patients treated with daraxonrasib and 31.7% of those receiving chemotherapy remained progression-free in the *RAS G12* population, compared with 56.0% and 32.9%, respectively, in the overall population [18].

Daraxonrasib also increased the likelihood of achieving an objective response. In the *RAS G12* population, the ORR was 33.2% (95% CI, 27.0–39.9) with daraxonrasib compared with 11.8% (95% CI, 7.8–16.8) with chemotherapy. In the overall population, the corresponding ORRs were 31.6% (95% CI, 25.8–38.0) and 11.2% (95% CI, 7.5–15.9), respectively [18].

Clinical benefits also included patient-reported outcomes. In the RAS G12 population, the median time to deterioration in pain was 9.0 months with daraxonrasib and 3.7 months with chemotherapy (HR, 0.51; 95% CI, 0.37–0.71; *p* < 0.001). Similar results were observed in the overall population, with corresponding median times of 9.2 and 3.8 months (HR, 0.51; 95% CI, 0.37–0.71; *p* < 0.001). The median time to deterioration in global health status–quality of life was 5.6 months with daraxonrasib and 2.4 months with chemotherapy in the *RAS G12* population (HR, 0.60; 95% CI, 0.45–0.80; *p* < 0.001), and 5.7 versus 2.6 months in the overall population (HR, 0.60; 95% CI, 0.46–0.79; *p* < 0.001) [18]. These findings indicate that the improvement in disease control was accompanied by a delayed deterioration in clinically relevant patient-reported outcomes.

### 3.5. Interpretation of Results

The results of the RMC-6236-001 [19] and RASolute 302 [18] studies are consistent with one another. In the cohort of patients with the *RAS G12* mutation receiving 300 mg of daraxonrasib in the second-line setting in a Phase 1–2 trial, the ORR was 35%, the median PFS was 8.5 months, and the median OS was 13.1 months [19]. In a randomized Phase 3 trial, these values were 33.2%, 7.3 months, and 13.2 months, respectively [18]. Similar results obtained in an exploratory study and in a controlled Phase 3 trial reinforce the credibility of the observed antitumor activity [18,19].

The most important outcome of RASolute 302 was an approximately twofold increase in median OS and PFS compared with active chemotherapy [18]. Of particular significance is the 60% reduction in the hazard of death in the population of patients previously treated for metastatic PDAC, for whom existing treatment options had provided limited efficacy [18]. The survival benefit was accompanied by an ORR that was nearly three times higher and a subsequent improvement in pain and quality of life [18].

The strongest and confirmatory evidence from RASolute 302 pertains to the prespecified primary efficacy population of patients with *RAS G12* mutations, in whom OS and PFS constituted the dual primary endpoints [18]. In contrast, analyses of patients without *RAS G12* mutations and of individual rare molecular subgroups were exploratory and were not powered for formal hypothesis testing. Among the 41 patients without *RAS G12* mutations, the OS analysis suggested a possible treatment benefit, whereas the PFS analysis did not demonstrate an advantage over chemotherapy [18]. These estimates were based on small numbers of patients and events and were characterized by wide confidence intervals, resulting in substantial statistical uncertainty. Accordingly, the findings in the non-*RAS G12* population should be regarded as descriptive and hypothesis-generating only and should not be interpreted as evidence of efficacy in any specific rare *RAS* variant. Additional prospective data with adequately represented molecular subgroups are required before conclusions regarding efficacy beyond the *RAS G12* population can be drawn [18].

When interpreting the results, the open-label nature of both studies should also be taken into account [18,19]. In RASolute 302, the lack of blinding may have influenced the reporting of adverse events, decisions regarding treatment modifications, and the assessment of patient-reported endpoints, although PFS and radiological response were assessed centrally in a blinded manner [18]. 15.1% of patients assigned to chemotherapy did not initiate treatment, compared with 2.8% of patients assigned to daraxonrasib [18]. Furthermore, the reported OS results were derived from the first planned interim analysis, with a median follow-up of 8.5 months [18]. Only patients in good performance status (ECOG 0–1) were enrolled in the study, which may limit the direct generalizability of the results to patients in poorer general condition [18,19].

In summary, daraxonrasib demonstrated clinically and statistically significant superiority over standard chemotherapy in terms of OS, PFS, ORR, time to pain progression, and quality of life in previously treated patients with metastatic PDAC [18]. The results suggest that broad inhibition of the active form of *RAS* may constitute an effective therapeutic strategy in a cancer in which the *RAS* pathway is a major driver of disease progression [18,19]. The current evidence is particularly compelling for the population with *RAS G12* mutations, whereas efficacy in rarer *RAS* variants and in tumors without an identified mutation requires further evaluation [18,19].

### 3.6. Toxicity Profile

In the Phase I/II RMC-6236-001 trial, the safety and tolerability of daraxonrasib administered as monotherapy at various doses ranging from 10 mg to 400 mg were evaluated in patients with previously treated, advanced PDAC harboring a *RAS* mutation. Daraxonrasib exhibited an acceptable, predictable, and clinically manageable toxicity profile [19].

Treatment-related adverse events (TRAEs) of any grade occurred in 96% of all enrolled patients regardless of dose, while TRAEs of grade 3 or higher (grade ≥ 3) were reported in 30% of patients [19]. Toxicity occurred more frequently at higher doses (≤120 mg [88%], 160–220 mg [100%], 300 mg [96%]). The 300-mg once-daily dose was subsequently selected as the optimal dose for the Phase III trial and demonstrated a favorable benefit-risk ratio [19]. The maximum tolerated dose (MTD) was not formally reached; however, the use of a 400-mg dose was associated with a higher frequency of dose modifications, which limited the ability to maintain continuous treatment. Therefore, the optimal dose of 300 mg was selected for further studies precisely because of its better tolerability and the possibility of long-term, uninterrupted administration compared to the 400 mg dose [19].

The mechanism-related adverse event profile primarily included skin symptoms (88%) and gastrointestinal complaints, such as diarrhea (46%), nausea (42%), and vomiting (31%). However, in most cases, cutaneous and gastrointestinal toxicity was mild or moderate (Grade 1 or 2). Other common adverse effects included mucositis and/or stomatitis, fatigue, paronychia, decreased appetite, and dry skin [19].

Discontinuation of the drug due to TRAE was extremely rare and occurred in only 1 patient (<1%). Dosing modifications (reduction or interruption), on the other hand, affected a large proportion of patients, particularly those receiving the 300 mg dose; however, most of them were able to safely resume treatment while maintaining a high relative dose intensity [19].

In the Phase III RASolute 302 trial, the safety profile of daraxonrasib as monotherapy was evaluated in comparison with standard chemotherapy selected by the investigator. Daraxonrasib also exhibited an acceptable toxicity profile distinct from that of conventional cytostatic agents [18].

TRAEs of any grade were reported in 97.9% of patients receiving daraxonrasib and in 93.5% of patients in the chemotherapy arm. Although the overall incidence of adverse events of any grade is high, drug-related grade 3 or higher events occurred less frequently than in the chemotherapy group (43.6% vs. 57.5%) [18].

The adverse event profile stems primarily from the specific nature of RAS pathway blockade and was characterized mainly by the presence of cutaneous and gastrointestinal symptoms. The most common adverse events were rash (85.5%), diarrhea (58.1%), nausea (46.5%), and vomiting (36.9%), stomatitis (53.1%), and fatigue (23.2%). Daraxonrasib was also associated with a reduction in severe organ- and system-related complications characteristic of chemotherapy. A significantly lower incidence of bone marrow suppression was observed, primarily neutropenia (7.5% vs. 38.3%), anemia (18.3% vs. 39.7%), and peripheral neuropathy (1.7% vs. 25.2%) [18].

This drug demonstrated better clinical tolerability, which resulted in a reduced need for dose modifications and less frequent discontinuation of therapy due to toxicity. The need for dose reduction was significantly lower in the daraxonrasib arm (36.1%) than in the chemotherapy arm (57.5%), while permanent discontinuation of treatment due to TRAE occurred in only 1.2% of patients in the daraxonrasib group compared with 11.2% in the chemotherapy group [18].

In the Phase III RASolute 302 trial, one case of fatal treatment-related pneumonitis was reported. Despite this incident, pneumonia remains a rare adverse event [18].

Overall, the safety profile of daraxonrasib proved consistent and highly comparable across both trials. Minor numerical variations in the incidence of specific adverse events are referable to the significantly larger cohort in the Phase III trial (*N* = 248 vs. *N* = 83), variations in exposure duration, and the implementation of proactive dermatologic and gastrointestinal prophylactic measures in the Phase III setting. Both studies consistently demonstrated that toxicity is predominantly Grade 1 or 2, highly manageable through standard supportive care, and rarely leads to permanent drug discontinuation [18,19].

The marked improvement in the tolerability profile led to a prolongation of the time to functional deterioration and an improvement in patients’ quality of life. The time to deterioration, including worsening of cancer pain and death, was 9.2 months in the daraxonrasib group versus 3.8 months in the chemotherapy group. The median time to deterioration in overall health and quality of life (QoL) was 5.7 months versus 2.6 months, favoring daraxonrasib [18].

The overall safety profile of the drug and the incidence of adverse events associated with daraxonrasib treatment, compared with chemotherapy, are summarized in Table 4 and Table 5.

## 4. Discussion

This systematic review identified two clinical studies evaluating daraxonrasib in previously treated advanced or metastatic PDAC: the single-arm Phase 1–2 RMC-6236-001 study and the randomized Phase III RASolute 302 trial [18,19]. Taken together, the available evidence supports clinically meaningful antitumor activity of multiselective RAS(ON) inhibition in this setting. The activity observed in the early-phase study was subsequently supported by randomized evidence from RASolute 302, in which daraxonrasib significantly improved OS and PFS compared with investigator-selected chemotherapy in the prespecified RAS G12 population [18]. Importantly, these survival benefits were accompanied by a higher ORR and delayed deterioration in pain and global health status–quality of life, suggesting that improved disease control was accompanied by clinically relevant patient-reported benefit.

The interpretation of these findings nevertheless requires consideration of the limitations of the available evidence. The strongest evidence applies to patients with *RAS G12*-mutated PDAC, whereas data for less common *RAS* variants remain limited and exploratory [20,21]. In addition, RMC-6236-001 was a single-arm early-phase study, precluding causal comparisons between treatment-line subgroups, while RASolute 302 was open-label and enrolled predominantly patients with good performance status [18,19]. The relatively short follow-up of the Phase III trial and the small number of patients in rare molecular subgroups further limit conclusions regarding long-term efficacy and activity beyond the *RAS G12* population. Nevertheless, the consistency of the efficacy signal across the two studies, together with the distinct and generally manageable toxicity profile observed with daraxonrasib, supports further evaluation of RAS(ON) inhibition as a clinically relevant strategy in PDAC.

To place these findings in the broader context of precision oncology in PDAC, other biomarker-directed strategies are briefly considered below. Pembrolizumab and PARP inhibitor-based approaches are discussed solely as examples of molecularly selected treatment strategies and not as direct therapeutic comparators to daraxonrasib. Daraxonrasib and pembrolizumab represent distinct biomarker-directed therapeutic strategies in PDAC. Current clinical evidence for daraxonrasib is strongest in *RAS G12*-mutated disease, whereas pembrolizumab is relevant primarily to the rare MSI-H/dMMR subgroup [18,20]. These therapies address fundamentally different molecular populations and should not be regarded as competing treatment options. Moreover, differences in molecular selection, study design, population size, and therapeutic setting preclude meaningful cross-trial comparisons of efficacy outcomes. The distinct molecular and clinical contexts of these strategies are summarized in Table 6.

A key difference between daraxonrasib and pembrolizumab lies in the potential molecular scope of the two strategies. *RAS* oncogenic mutations are present in over 90% of PDAC cases, making this pathway a potential therapeutic target for a significant proportion of patients [18,19]. In contrast, dMMR is rare; in an analysis by Hu et al. involving 833 cases of PDAC, it was found in only 7 patients (0.8%) [21]. The limited prevalence of this biomarker significantly narrows the population potentially eligible for pembrolizumab based on MSI-H/dMMR. At the same time, in appropriately selected patients, the response to immunotherapy can be exceptionally durable. In the updated analysis of KEYNOTE-158, which included 373 patients with various advanced MSI-H/dMMR cancers, the median duration of response to pembrolizumab was 63.2 months with a 4.5-year follow-up period [22]. However, this result applies to the entire population of non-colorectal cancers and should not be directly extrapolated to PDAC. The potential for durable disease control in pancreatic cancer is supported by retrospective data from the Mayo Clinic, in which patients with dMMR/MSI-H treated with immune checkpoint inhibitors in a palliative setting had an ORR of 75%, including 20% complete responses, and the median time to progression had not yet been reached [23]. Given the small sample size and retrospective nature of this cohort, these results should be interpreted with caution. The available data thus point to a different profile of potential benefit for the two strategies. Targeting RAS gives daraxonrasib a potentially much broader molecular scope than selection based on MSI-H/dMMR, although current clinical evidence is strongest for the population with *RAS G12* mutations [18,19]. In contrast, pembrolizumab is used in the much rarer MSI-H/dMMR population, in which some patients may achieve particularly long-lasting responses [21,22,23].

Other biomarker-directed approaches, including PARP inhibitor-based maintenance strategies, further illustrate the molecular heterogeneity of PDAC. The POLAR trial evaluated pembrolizumab combined with olaparib after disease control with platinum-based chemotherapy, whereas POLO evaluated olaparib maintenance in *germline BRCA1/2*-mutated PDAC [24,25,26]. These studies involved different molecularly selected populations, treatment settings, and therapeutic objectives and are therefore mentioned here only as contextual examples rather than as direct comparators to daraxonrasib. Caution is warranted when interpreting evidence across biomarker-directed strategies in PDAC because the relevant studies differ substantially in molecular selection, study design, and therapeutic setting. In particular, RASolute 302 evaluated daraxonrasib in previously treated metastatic PDAC, whereas pembrolizumab data in PDAC are derived primarily from a small MSI-H/dMMR subgroup within the single-arm Phase II KEYNOTE-158 study [18,20]. These therapies address distinct molecularly defined populations and should not be considered directly comparable.

A comparative analysis of the safety profiles of daraxonrasib, standard chemotherapy, and pembrolizumab reveals fundamental differences in the mechanisms of action of these drugs, which translate into different toxicity profiles. The toxicity profile of daraxonrasib (RMC-6236) is described by the study authors primarily as mild adverse events, graded as Grade 1 or 2 on the CTCAE scale. These were primarily dermatological and gastrointestinal in nature, which is a direct consequence of the inhibition of the MAPK pathway in these tissues. Although nearly all patients (96–97.9%) experienced some form of toxicity, this drug is significantly better tolerated than standard chemotherapy, resulting in less frequent treatment discontinuation due to adverse effects [18,19]. The most common TRAE was rash. The next most common adverse effects were diarrhea, stomatitis or mucositis, nausea, vomiting, and fatigue [18,19]. The most common treatment-related serious adverse events leading to hospitalization were gastrointestinal complications in both the daraxonrasib group (4.1%) and the chemotherapy group (5.6%). Less common but clinically significant adverse events associated with daraxonrasib also included paronychia, anemia, decreased appetite, dry skin, elevated transaminase levels, and asthenia [18,19].

Toxicity management relies primarily on routine clinical interventions and dosage adjustments, including temporary treatment interruptions or dose reductions. The median cumulative duration of treatment interruptions due to TRAEs was 16 days [21]. Despite the need to modify the dosing regimen, most patients were able to resume therapy at the same or a reduced dose [18]. The decision to reduce or temporarily suspend the dose was based on the severity of symptoms according to the CTCAE scale [20,21]. Compared to standard chemotherapy, daraxonrasib exhibits a more favorable tolerability profile, which is primarily reflected in a significantly lower incidence of hematologic and neurological toxicity-side effects that, with traditional cytostatic agents, often lead to myelosuppression or persistent neuropathy [18,19].

Pembrolizumab presents a different profile; when used as monotherapy in the KEYNOTE-158 trial, it caused adverse events in 64.8% of patients with MSI-H tumors, with 14.6% experiencing Grade 3 or higher events. The most common adverse events were fatigue (14.6%), pruritus (12.9%), diarrhea (12.0%), and asthenia (10.7%). However, the key risk associated with its use is immune-related adverse events (irAE), including autoimmune pneumonia, intestinal inflammation, liver inflammation, and endocrine disorders such as hypothyroidism. In this study, they occurred in 23.2% of patients, of whom 6.0% experienced serious Grade 3 or 4 irAE [20].

In summary, future research should focus on optimizing strategies for the prevention and management of dermatologic toxicity, which is crucial for maintaining the quality of life of patients treated with *RAS* inhibitors [18,19,26,27]. Given the prolongation of the median OS to 13.2 months with daraxonrasib, it is also essential to monitor cumulative toxicity resulting from long-term exposure to this drug. Long-term inhibition of the MAPK pathway may reveal additional complications not observed during short-term follow-up; therefore, further studies with extended follow-up periods are necessary [28].

In the context of future studies, it is also very important to evaluate potential interactions in new regimens combining *RAS* inhibitors with immunotherapy. This is particularly important with regard to gastrointestinal complications, which are common to both types of drugs [18,19,20,29]. Distinguishing between the non-immunological and immunological etiologies of these adverse events will also require the development of additional diagnostic and therapeutic tests [29,30]. Researchers should aim to identify predictive biomarkers for severe immune-mediated adverse reactions. This would help minimize the risk of treatment discontinuation in patients with the rare MSI-H/dMMR subgroup [31].

A further important limitation of this review pertains to its methodological approach to evidence synthesis. Given the substantial heterogeneity in study design between the single-arm Phase I/II RMC-6236-001 trial and the randomized Phase III RASolute 302 trial, a quantitative meta-analysis was not performed, and findings were instead synthesized narratively, in accordance with PRISMA 2020 and Cochrane Handbook recommendations against pooling data from methodologically disparate study designs. While this approach preserves the internal validity of each individual finding, it inherently limits the precision and generalizability of the overall conclusions: unlike a pooled meta-analytic estimate, a narrative synthesis cannot provide a single quantitative effect size, cannot formally assess between-study heterogeneity (e.g., via I^2^ statistics), and remains more susceptible to selective emphasis in the interpretation of results. Furthermore, with only two eligible studies identified, the evidence base for daraxonrasib in PDAC remains preliminary, and the conclusions of this review should be regarded as hypothesis-generating rather than definitive. As additional clinical trials of daraxonrasib and other RAS(ON) inhibitors are completed, future updates of this review incorporating a larger number of homogeneous studies will be necessary to permit formal quantitative synthesis and to strengthen the certainty of the evidence.

## 5. Conclusions

Innovative molecularly targeted therapies may offer new hope for the treatment of PDAC, given its resistance to conventional treatment regimens and poor prognosis. In this area, daraxonrasib (RMC-6236) represents a promising therapeutic option. As the first drug in its class, it is a non-covalent, multiselective pan-inhibitor of the active form of RAS(ON). Its unique mechanism of forming an intracellular tri-complex with CypA allows for the creation of a steric block, thereby preventing the oncogenic interaction of the active RAS-GTP protein with kinases of the RAF family. This leads to profound inhibition of the MAPK/ERK and AKT proliferative cascades. Additionally, daraxonrasib acts in a multiselective manner. It blocks a broad spectrum of oncogenic variants of three key isoforms, including the predominant codon-12 mutations. However, the strongest clinical evidence currently supports its use in patients with *RAS G12* mutations, while the efficacy of daraxonrasib in rarer *RAS* variants remains uncertain. Translational and preclinical studies reveal the drug’s strong anti-invasive potential, based on multiple mechanisms: inactivation of the AKT/ETS1 axis, suppression of MMP1/MMP9 metalloproteinases, the ability to stimulate specific CD4+/CD8+ anticancer immunity, and synergy with anti-PD-1 therapy. However, these findings should be considered as preclinical or translational evidence and should not be interpreted as clinical evidence of efficacy, as preclinical studies were not included in the systematic review. Significant and direct clinical validation of the efficacy of daraxonrasib as monotherapy at a dose of 300 mg daily was demonstrated in the Phase 3 controlled clinical trial RASolute 302. Use of the drug in patients with *RAS G12* mutations resulted in a twofold increase in OS from 6.6 to 13.2 months (HR = 0.40; *p* < 0.001) and in PFS from 3.5 to 7.3 months (HR = 0.45; *p* < 0.001) compared with conventional chemotherapy. The ORR increased nearly threefold (33.2% vs. 11.8%; *p* < 0.001), demonstrating full consistency and reproducibility with the results of the Phase 1–2 RMC-6236-001 trial. Importantly, prolonged tumor control was accompanied by a more than twofold delay in the time to progression of pain (9.0 vs. 3.7 months; HR = 0.51) and deterioration in patients’ overall health (5.6 vs. 2.4 months; HR = 0.60), which suggests a potential benefit in maintaining patients’ quality of life. Daraxonrasib is characterized by a predictable and clinically manageable tolerability profile. It exhibits a lower incidence of severe hematologic toxicities complications compared to the chemotherapy arm (neutropenia: 7.5% vs. 38.3%, anemia: 18.3% vs. 39.7%) and neurological complications (peripheral neuropathy: 1.7% vs. 25.2%). On-target adverse events resulting from the physiological blockade of the pathway, such as skin rash (85.5%), diarrhea (58.1%), and oral mucositis (53.1%), are predominantly mild or moderate in severity (Grade 1 or 2). This allowed for the safe continuation of treatment and resulted in a minimal rate of permanent treatment discontinuation due to toxicity (1.2% vs. 11.2%). Future studies should focus on improving the prevention of skin toxicity and evaluating complications associated with long-term inhibition of the MAPK cascade. Attention should also be given to the drug’s tolerability and its interactions in novel combination therapies that pair pan-*RAS* inhibitors with anti-PD-1 or FUS-based immunotherapy.

## 6. Limitations of the Review

### 6.1. Limitations of the Included Studies

Several limitations inherent to the two included studies must be considered when interpreting the evidence presented in this review.

The systematic review highlights the clinical efficacy and safety profile of daraxonrasib in the treatment of previously treated metastatic PDAC. Given the recent introduction and regulatory approval of daraxonrasib in clinical oncology, the current published clinical evidence still is limited to two main studies: the phase 1–2 RMC-6236-001 trial and the phase 3 RASolute 302 trial. The total number of published cohorts assessed to date remains relatively small, although both studies were conducted with a high degree of methodological rigour.

Methodological limitations also stem from the study designs used in the evidence reviewed. In the early phase of the RMC-6236-001 study, a non-randomised, single-arm design was used, which precludes direct statistical comparative inferences between dose cohorts or lines of treatment. Furthermore, both studies included in the review employed an open-label design. Although the primary radiological efficacy endpoints in the phase III trial, such as PFS and ORR, were assessed by a blinded, independent central review, the lack of blinding of investigators and patients may have influenced the reporting of adverse events, decisions regarding dose modification, and patient-reported quality-of-life outcomes.

Additional statistical limitations arise from the distribution of molecular subtypes within the study populations. Over 90% of the entire cohort exhibited RAS G12 mutations, whilst patients with rarer *RAS* variants, such as G13 or Q61 mutations, or those with the wild-type *RAS* gene, accounted for less than 10% of the total study population. The small sample size and limited number of events in these rare subgroups result in wide confidence intervals and high statistical uncertainty; consequently, the efficacy results in populations other than G12 are purely exploratory and serve to inform hypothesis formulation.

The data presented from the Phase III RASolute 302 trial reflect the first planned interim analysis, in which the median follow-up period was 8.5 months. It is necessary to continue long-term follow-up of patients as part of subsequent, mature analyses in order to fully determine the outcomes regarding long-term overall survival, potential late-onset adverse events, and the overall durability of response. Moreover, as the control arm of the Phase III trial used standard cytotoxic chemotherapy selected by the investigator, there are currently no direct clinical trials comparing daraxonrasib with other new targeted therapies or combination immunotherapies in subgroups of PDAC patients selected on the basis of biomarkers.

### 6.2. Limitations of the Present Review

This review is also subject to limitations arising from its own methodology. Given the significant differences in design between the single-arm Phase I/II trial RMC-6236-001 and the randomised Phase III trial RASolute 302, no quantitative meta-analysis was conducted. Instead, the results were summarised descriptively, in accordance with the PRISMA 2020 guidelines and the Cochrane Handbook, which advise against pooling data from studies with different methodological designs. Although this approach preserves the internal validity of individual findings. By its nature, by its nature limits precision and the ability to draw general conclusions. Unlike a pooled meta-analytic estimate, a narrative synthesis does not allow for the determination of a single quantitative effect size, does not permit a formal assessment of heterogeneity between studies and is more prone to the selective emphasis of certain aspects during the interpretation of results.

Furthermore, as a result of the systematic review, only two studies were identified that met the criteria. The evidence base regarding the use of daraxonrasib in PDAC remains preliminary, and the conclusions of this review should be regarded as a starting point for formulating hypotheses rather than as definitive. The search was restricted to studies published in English between January 2020 and July 2026, which may have resulted in the omission of relevant publications in other languages. Additionally, as with any systematic review, the possibility of publication bias favoring positive results cannot be ruled out. As further clinical trials on daraxonrasib and other RAS(ON) inhibitors are completed, it will be necessary to update this review in the future by incorporating a larger number of homogeneous studies, which will enable a formal quantitative synthesis to be carried out and will increase the certainty of the evidence.

## Figures and Tables

**Figure 1:**
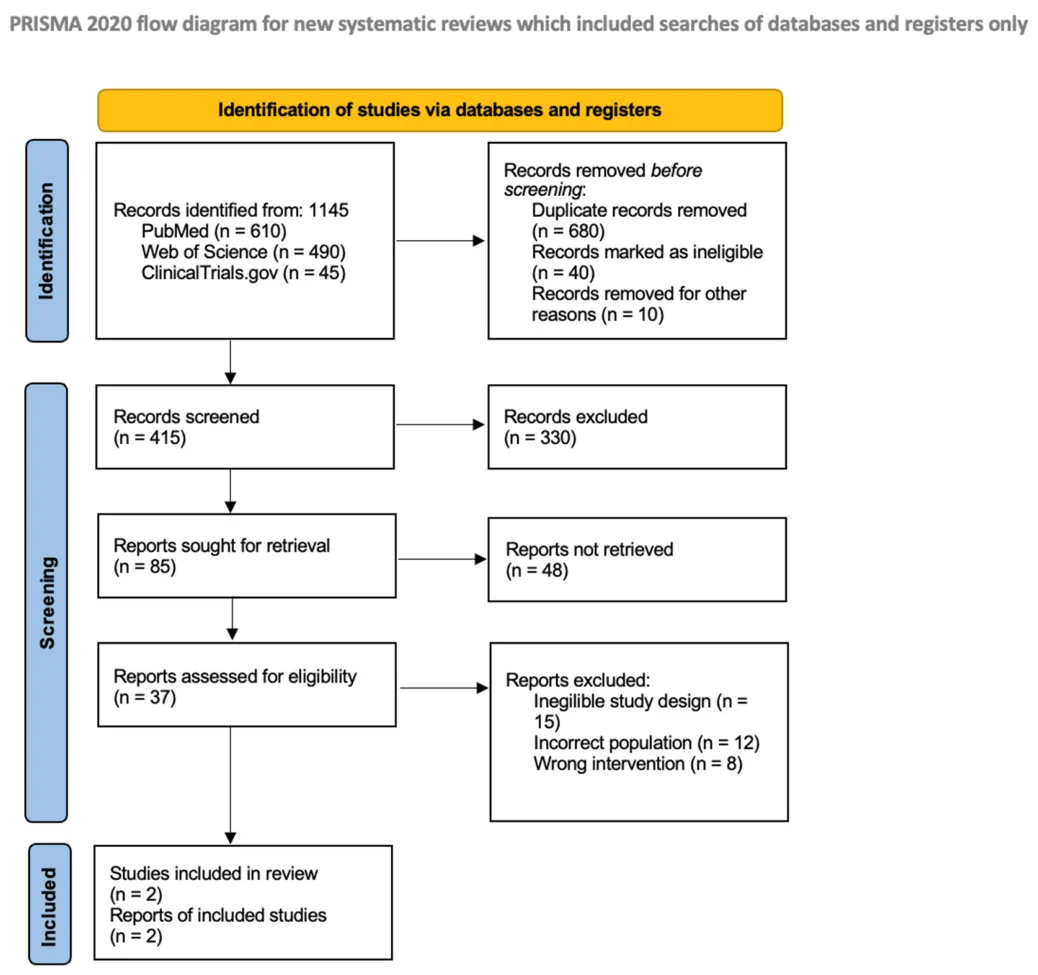
PRISMA flow diagram.

**Figure 2:**
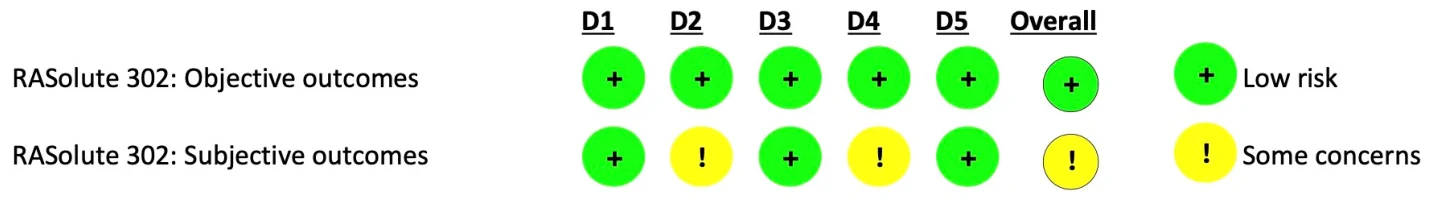
Risk of bias assessment for the included randomized controlled trial (RASolute 302) using the Cochrane RoB 2.0 tool.

**Figure 3:**
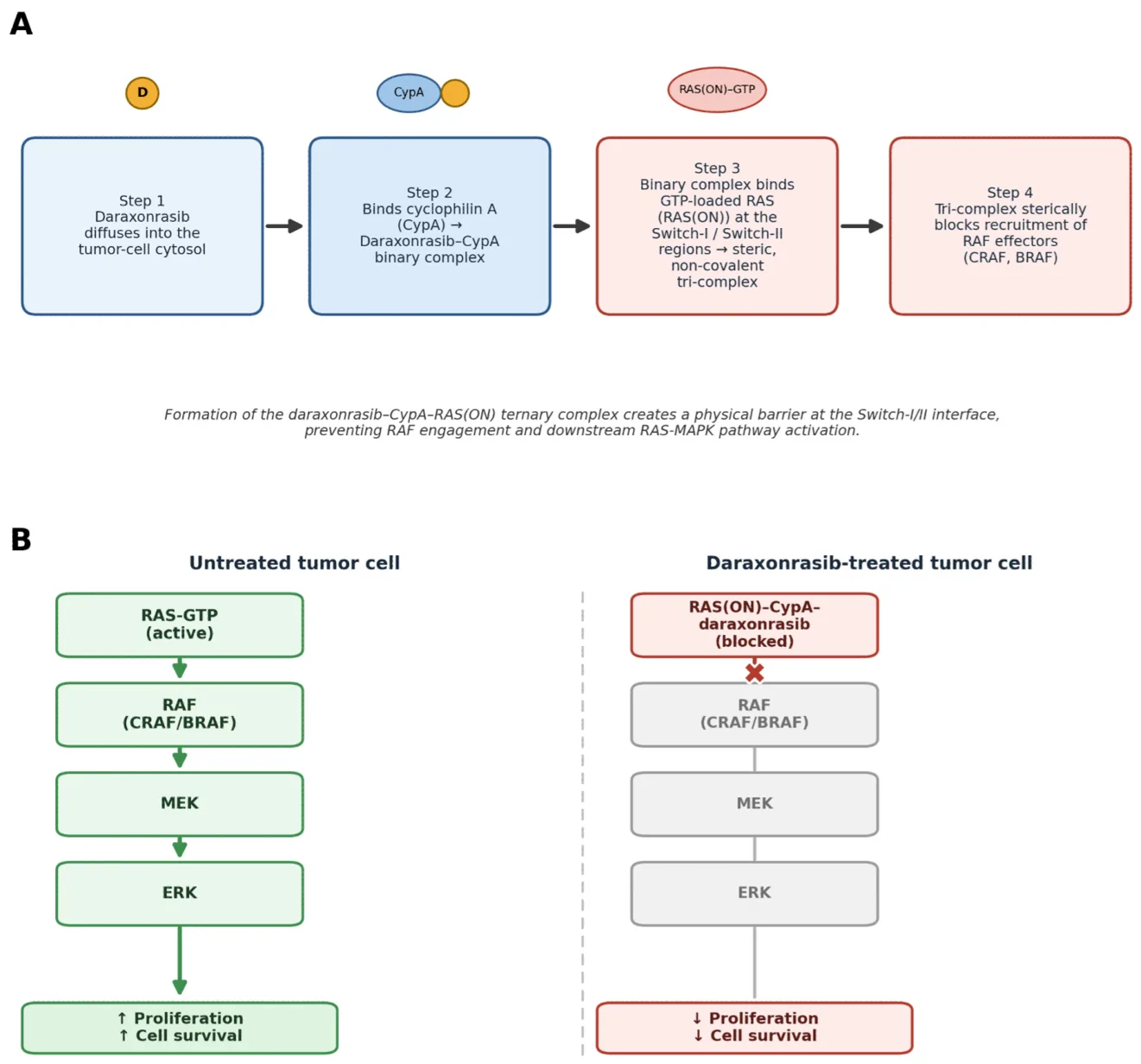
Mechanism of action of daraxonrasib. (**A**) Stepwise formation of the daraxonrasib–cyclophilin A (CypA)–RAS(ON) ternary complex. Daraxonrasib enters the tumor-cell cytosol and binds CypA to form a binary complex, which then binds GTP-loaded RAS (RAS(ON)) at the Switch-I/Switch-II interface, creating a steric barrier that blocks RAF (CRAF/BRAF) recruitment. Black arrows indicate the sequential order of complex assembly (Steps 1–4). (**B**) Functional consequence of tri-complex formation on downstream RAS–MAPK signaling. In untreated cells (left), active RAS-GTP drives the RAF–MEK–ERK cascade, promoting proliferation and survival; green arrows denote active signal transduction between pathway components. In daraxonrasib-treated cells (right), daraxonrasib-bound RAS is unable to engage RAF; the red cross indicates steric blockade of RAF recruitment at the RAS(ON)—CypA—daraxonrasib complex, and gray arrows indicate loss of downstream MEK and ERK activation as a consequence, resulting in suppressed MAPK pathway activity and reduced proliferative and survival signaling.

**Table 1:** Characteristics of clinical trials evaluating daraxonrasib in pancreatic ductal adenocarcinoma.

Study	Phase and Design	Study Population	Control Population	Intervention and Comparator	Primary Endpoints	Secondary Endpoints
RMC-6236-001; Wolpin et al. [19]	Phase 1–2, multicenter, open-label, single-arm dose-escalation and dose-optimization study conducted at 16 sites in the United States	168 patients with previously treated, advanced *RAS*-mutated PDAC; ECOG performance status 0–1; daraxonrasib administered as second- or later-line therapy	Not applicable	Daraxonrasib 10–400 mg orally once daily; the PDAC efficacy analysis included doses ≤300 mg; 300 mg once daily was selected as the recommended monotherapy dose	Safety and tolerability: TRAEs, Dose modifications, Recommended phase 3 dose	Efficacy: ORR, DCR, DoR, PFS, and OS Pharmacokinetics: AUC, Tmax, t1/2
RASolute302; O’Reilly et al. [18]	Phase 3, international, open-label, randomizedcontrolled trial conducted at 59 sites in six countries; 1:1 randomization	248 patients (241 received at least one dose of the drug); 228 patients (91.9%) had the *RAS G12* mutation (the *RAS G12* subpopulation); 20 patients had other mutations (G13, Q61) or no identifiable RAS mutation (including 9 patients without a *RAS* mutation).	252 patients (214 received at least one dose); 231 patients (91.7%) had a *RAS G12* mutation; 21 patients had other mutations or no identifiable *RAS* mutation (including 7 patients with no *RAS* mutation).	Daraxonrasib 300 mg orally once daily versus investigator’s choice of chemotherapy	OS and PFS in the *RAS* G12 population	OS and PFS in the overall population, ORR, and patient-reported quality-of-life outcomes, TRAEs

Abbreviations: AUC, area under the curve; DCR, disease control rate; DoR, duration of response; ECOG, Eastern Cooperative Oncology Group; mPDAC, metastatic pancreatic ductal adenocarcinoma; ORR, objective response rate; OS, overall survival; PDAC, pancreatic ductal adenocarcinoma; PFS, progression-free survival; t1/2, elimination half-life; Tmax, time to maximum concentration; TRAE, treatment-related adverse events.

**Table 2:** Clinical efficacy of daraxonrasib 300 mg according to treatment line and *RAS* mutation status in the RMC-6236-001 trial.

Efficacy Outcome	Second-Line Therapy, *RAS G12*-Mutated (*n* = 26)	Second-Line Therapy, All *RAS*-Mutated (*n* = 38)	Third- or Later-Line Therapy, *RAS G12*-Mutated (*n* = 38)	Third- or Later-Line Therapy, All *RAS*-Mutated (*n* = 45)
Objective response, *n* (%)	9 (35)	11 (29)	8 (21)	9 (20)
ORR, % (95% CI)	35 (17–56)	29 (15–46)	21 (10–37)	20 (10–35)
Complete response, *n* (%)	1 (4)	1 (3)	0	0
Partial response, *n* (%)	8 (31)	10 (26)	8 (21)	9 (20)
Stable disease, *n* (%)	15 (58)	25 (66)	25 (66)	29 (64)
Progressive disease, *n* (%)	1 (4)	1 (3)	4 (11)	5 (11)
Not evaluable, *n* (%)	1 (4)	1 (3)	1 (3)	2 (4)
Disease control, *n* (%)	24 (92)	36 (95)	33 (87)	38 (84)
DCR, % (95% CI)	92 (75–99)	95 (82–99)	87 (72–96)	84 (71–94)
Median DoR, months (95% CI)	8.2 (3.8–NE)	8.2 (3.8–8.8)	3.5 (2.9–NE)	3.3 (2.8–NE)
Median PFS, months (95% CI)	8.5 (6.7–10.5)	8.1 (5.9–10.1)	4.3 (4.1–8.1)	4.3 (4.1–7.8)
Median OS, months (95% CI)	13.1 (10.9–NE)	15.6 (10.9–NE)	8.6 (6.4–11.9)	9.0 (6.7–11.1)

Abbreviations: CI, confidence interval; DCR, disease control rate; DoR, duration of response; NE, not evaluable; ORR, objective response rate; OS, overall survival; PFS, progression-free survival. Notes: All *RAS*-mutated groups included tumors harboring *RAS* mutations at codons G12, G13, or Q61. Objective response was defined as a confirmed, complete or partial response. Disease control was defined as a complete response, partial response, or stable disease. Second-line therapy was defined as treatment following one previous line of therapy for metastatic disease or following neoadjuvant or adjuvant therapy if disease progression occurred within 6 months.

**Table 3:** Clinical efficacy of daraxonrasib versus chemotherapy in the phase 3 RASolute 302 trial.

Population and Efficacy Outcome	Daraxonrasib	Chemotherapy	HR (95% CI)	*p* Value
*RAS G12* population: daraxonrasib *n* = 228; chemotherapy *n* = 231				
Median OS, months (95% CI)	13.2 (10.0–NR)	6.6 (5.4–8.2)	0.40(0.30–0.54)	<0.001
Median PFS, months (95% CI)	7.3 (6.3–8.1)	3.5 (2.9–3.8)	0.45 (0.34–0.59)	<0.001
ORR, % (95% CI)	33.2 (27.0–39.9)	11.8 (7.8–16.8)	N/A	NR
Median time to pain deterioration, months	9.0	3.7	0.51 (0.37–0.71)	<0.001
Median time to deterioration in global health status–quality of life, months	5.6	2.4	0.60 (0.45–0.80)	<0.001
Overall population: daraxonrasib *n* = 248; chemotherapy *n* = 252				
Median OS, months (95% CI)	13.2 (10.0–NR)	6.7 (5.8–8.0)	0.40 (0.30–0.53)	<0.001
Median PFS, months (95% CI)	7.2 (5.7–7.5)	3.6 (2.9–4.2)	0.49 (0.38–0.64)	<0.001
ORR, % (95% CI)	31.6 (25.8–38.0)	11.2 (7.5–15.9)	N/A	NR
Median time to pain deterioration, months	9.2	3.8	0.51 (0.37–0.71)	<0.001
Median time to deterioration in global health status–quality of life, months	5.7	2.6	0.60 (0.46–0.79)	<0.001

Abbreviations: CI, confidence interval; EORTC QLQ-C30, European Organisation for Research and Treatment of Cancer Quality of Life Questionnaire–Core 30; EORTC QLQ-PAN26, European Organisation for Research and Treatment of Cancer Quality of Life Questionnaire–Pancreatic Cancer Module; HR, hazard ratio; N/A, not applicable; NR, not reported in the primary publication. ORR, objective response rate; OS, overall survival; PFS, progression-free survival; RECIST, Response Evaluation Criteria in Solid Tumors.

**Table 4:** Treatment-related adverse events (TRAEs) and safety profile across study cohorts.

Event Category	Daraxonrasib 300 mg (*N* = 83) [19]	Daraxonrasib 300 mg (*N* = 248) [18]	Chemotherapy (*N* = 252) [18]
TRAE adverse events of any grade	96%	97.9%	93.5%
Adverse events: Severe TRAE (grade ≥ 3)	34%	43.6%	57.5%
Serious Adverse Events (SAE)	6%	10.8%	18.7%
Dose reduction due to TRAE	30%	36.1%	57.5%
Dose interruption due to TRAE	43%	56%	55.1%
Permanent treatment discontinuation due to TRAE	0%	1.2%	11.2%

Abbreviations: N—Number of patients in the cohort, SAE—Serious Adverse Event, TRAE—Treatment-Related Adverse Event.

**Table 5:** Incidence of specific adverse events across treatment groups.

Side Effect	Daraxonrasib 300 mg [19]	Daraxonrasib 300 mg [18]	Chemotherapy [18]
Rash	90%	85.5%	5.6%
Diarrhea	52%	58.1%	37.9%
Stomatitis	54%	53.1%	17.3%
Nausea	39%	46.5%	39.3%
Vomiting	36%	36.9%	22.0%
Fatigue	17%	23.2%	44.4%
Anemia	not mentioned	18.3%	39.7%
Neutropenia	not mentioned	7.5%	38.3%
Peripheral neuropathy	not mentioned	1.7%	25.2%

**Table 6:** Distinct molecular and clinical contexts of daraxonrasib and pembrolizumab in pancreatic ductal adenocarcinoma.

Parameter	Daraxonrasib (RMC-6236)	Pembrolizumab
Key clinical study	RASolute 302 [18]	KEYNOTE-158 [20]
Study design	Phase III, international, open-label, randomized controlled trial	Phase II, multicohort, open-label, single-arm basket trial
Clinical setting	Previously treated metastatic PDAC	Previously treated advanced MSI-H/dMMR noncolorectal solid tumors; pancreatic cancer subgroup
Population represented in the cited clinical evidence	*RAS G12* population: *n* = 459; daraxonrasib *n* = 228, chemotherapy *n* = 231	MSI-H/dMMR pancreatic cancer subgroup: *n* = 22
Biomarker/molecular selection	*RAS G12* mutation for the primary efficacy population	MSI-H and/or dMMR
Biomarker prevalence in PDAC	Oncogenic *RAS* mutations occur in >90% of PDAC; however, the strongest confirmatory clinical evidence for daraxonrasib currently pertains to *RAS G12*-mutated disease [20,21].	dMMR was identified in 0.8% (7/833) of PDAC cases in the cohort reported by Hu et al. [21].
Mechanism of action	Oral multiselective RAS(ON) tri-complex inhibitor targeting the active, GTP-bound state of mutant and wild-type RAS	Anti-PD-1 monoclonal antibody restoring antitumor T-cell activity
Treatment regimen in the cited study	300 mg orally once daily	200 mg intravenously every 3 weeks
Evidence base in pancreatic cancer	Randomized phase III trial with active chemotherapy comparator; OS and PFS as dual primary endpoints in the *RAS G12* population	Phase II tumor-agnostic basket trial; pancreatic cancer subgroup *n* = 22; no pancreatic cancer-specific control arm

Abbreviations: dMMR, mismatch repair deficiency; GTP, guanosine triphosphate; MSI-H, microsatellite instability-high; OS, overall survival; PD-1, programmed cell death protein 1; PDAC, pancreatic ductal adenocarcinoma; PFS, progression-free survival; *RAS*, rat sarcoma virus family of small GTPases. Note: This table is intended to illustrate the distinct molecular and clinical contexts of these therapies rather than to provide a comparative efficacy assessment. Daraxonrasib and pembrolizumab target different biomarker-defined patient populations, and cross-trial comparisons of efficacy outcomes are not methodologically appropriate.

## Data Availability

No new data were created or analyzed in this study. Data sharing is not applicable to this article.

## Supplementary Materials

The following supporting information can be downloaded at: https://www.mdpi.com/article/10.3390/cancers18193112/s1, Table S1: Full database-specific search strategies for PubMed, Web of Science, ClinicalTrials.gov, and Scopus; Table S2: Domain-level rationale and supporting judgments for the outcome-specific Cochrane Risk of Bias 2 (RoB 2) assessment of the RASolute 302 trial; Table S3: Methodological Index for Non-Randomized Studies (MINORS) assessment of the single-arm RMC-6236-001 trial; PRISMA 2020 Checklist [32].

## Abbreviations

5-FU	Fluorouracil
CA 19-9	Carbohydrate antigen 19-9
CD4+/CD8+	Cluster of differentiation 4/8 (T-lymphocyte subsets)
CE-CT	Contrast-enhanced computed tomography
CI	Confidence interval
COX-2	Cyclooxygenase-2
CR	Complete response
CRC	Colorectal cancer
CTCAE	Common Terminology Criteria for Adverse Events
CXCR4	C-X-C chemokine receptor type 4
CypA	Cyclophilin A
DCR	Disease control rate
dMMR	Mismatch repair deficiency
DoR	Duration of response
ECOG	Eastern Cooperative Oncology Group
EORTC QLQ-C30	European Organisation for Research and Treatment of Cancer Quality of Life Questionnaire–Core 30
EORTC QLQ-PAN26	European Organisation for Research and Treatment of Cancer Quality of Life Questionnaire–Pancreatic Cancer Module
ERK	Extracellular signal-regulated kinase
EUS	Endoscopic ultrasound
FDA	Food and Drug Administration
FOLFIRINOX	Folinic acid, fluorouracil, irinotecan, and oxaliplatin (chemotherapy regimen)
FUS/HIFU	Focused ultrasound/high-intensity focused ultrasound
GLOBOCAN	Global Cancer Observatory (global cancer statistics database)
GTP	Guanosine triphosphate
HR	Hazard ratio
HRD	Homologous recombination deficiency
IC50	Half-maximal inhibitory concentration
irAE	Immune-related adverse event
ITT	Intention-to-treat
MAPK	Mitogen-activated protein kinase
MDSCs	Myeloid-derived suppressor cells
mITT	Modified intention-to-treat
MMP	Matrix metalloproteinase
mPDAC	Metastatic pancreatic ductal adenocarcinoma
MSI-H	Microsatellite instability-high
MSS	Microsatellite-stable
MTD	Maximum tolerated dose
N	Number of patients (cohort size)
NALIRIFOX	Liposomal irinotecan, fluorouracil, leucovorin, and oxaliplatin (chemotherapy regimen)
NAT	Neoadjuvant chemotherapy
ncHRD	Non-core homologous recombination deficiency
NCRT	Neoadjuvant chemoradiotherapy
NE	Not evaluable
NF-κB	Nuclear factor kappa-light-chain-enhancer of activated B cells
NIH	National Institutes of Health
NR	Not reached/not reported
NSCLC	Non-small cell lung cancer
ORR	Objective response rate
OS	Overall survival
PARP	Poly(ADP-ribose) polymerase
PASC	Pancreatic adenocarcinoma with squamous differentiation
PD	Progressive disease
PDAC	Pancreatic ductal adenocarcinoma
PD-1	Programmed cell death protein 1
PFS	Progression-free survival
PICO	Population, Intervention, Comparison, Outcome
PK	Pharmacokinetic(s)
PK/PD	Pharmacokinetic-pharmacodynamic
PR	Partial response
PRISMA	Preferred Reporting Items for Systematic Reviews and Meta-Analyses
PROSPERO	International Prospective Register of Systematic Reviews
QoL	Quality of life
R0	Microscopically complete resection (negative margin)
RCT	Randomized controlled trial
RECIST	Response Evaluation Criteria in Solid Tumors
RoB 2.0	Cochrane Risk of Bias tool for randomized trials, version 2.0
SAE	Serious adverse event
SD	Stable disease
Sp1	Specificity protein 1
TAMs	Tumor-associated macrophages
TME	Tumor microenvironment
Tmax	Time to maximum plasma concentration
TRAE	Treatment-related adverse event
Tregs	Regulatory T cells
