# Supplementary material for: Efficacy and Safety of Daraxonrasib (RMC-6236) in Previously Treated Metastatic RAS-Mutated Pancreatic Ductal Adenocarcinoma: A Systematic Review
Source: Cancers (Basel). 2026 Sep 25;18(19):3112. doi: 10.3390/cancers18193112 (PMC13664388; doi:10.3390/cancers18193112)
Supplement: Supplementary file 1 [file cancers-18-03112-s001.zip › Table S1.pdf]

Database	Exact Search String / Query Strategy	Filters & Limits Applied	Date Restrictions	Records Retrieved
**PubMed**	(("Daraxonrasib"[Title/Abstract] OR "RMC-6236"[Title/Abstract] OR "RMC 6236"[Title/Abstract] OR "RAS inhibitor"[Title/Abstract] OR "pan-RAS inhibitor"[Title/Abstract]) AND ("pancreatic ductal adenocarcinoma"[Title/Abstract] OR "pancreatic cancer"[Title/Abstract] OR "PDAC"[Title/Abstract] OR "Pancreatic Neoplasms"[Mesh]))	English language	Jan 1, 2020 – Jul 31, 2026	610
**Web of Science** (Core Collection)	TS=(("Daraxonrasib" OR "RMC-6236" OR "RMC 6236" OR "RAS inhibitor" OR "pan-RAS inhibitor") AND ("pancreatic ductal adenocarcinoma" OR "pancreatic cancer" OR "PDAC"))	English language, Topic search (TS)	Jan 1, 2020 – Jul 31, 2026	490
**ClinicalTrials.gov**	(Daraxonrasib OR RMC-6236 OR RMC 6236 OR RAS inhibitor) AND (Pancreatic Ductal Adenocarcinoma OR Pancreatic Cancer OR PDAC)	Interventional studies	First posted: Jan 1, 2020 – Jul 31, 2026	45
**Scopus**	TITLE-ABS-KEY(("Daraxonrasib" OR "RMC-6236" OR "RMC 6236" OR "RAS inhibitor" OR "pan-RAS inhibitor") AND ("pancreatic ductal adenocarcinoma" OR "pancreatic cancer" OR "PDAC"))	English language, Title/Abstract/Keyword search	Jan 1, 2020 – Jul 31, 2026	232 (0 additional eligible studies after deduplication)

Table S1. Full database-specific search strategies for PubMed, Web of Science, ClinicalTrials.gov, and Scopus.
