# Supplementary material for: Efficacy and Safety of Daraxonrasib (RMC-6236) in Previously Treated Metastatic RAS-Mutated Pancreatic Ductal Adenocarcinoma: A Systematic Review
Source: Cancers (Basel). 2026 Sep 25;18(19):3112. doi: 10.3390/cancers18193112 (PMC13664388; doi:10.3390/cancers18193112)
Supplement: Supplementary file 1 [file cancers-18-03112-s001.zip › Table S2.pdf]

RoB 2 Domain	Objective Outcomes (OS, BICR-assessed PFS)	Subjective & Safety Outcomes (AEs, PROs, Investigator PFS)	Supporting Rationale and Justification
**Domain 1: Risk of bias arising from the randomization process**	Low Risk	Low Risk	Centralized, computer-generated 1:1 randomization with adequate allocation concealment and balanced baseline clinical characteristics across arms.
**Domain 2: Risk of bias due to deviations from intended interventions**	Low Risk	Some concerns	Open-label trial design. While intention-to-treat (ITT) analysis was performed, lack of blinding could lead to differential treatment discontinuation or non-initiation prior to first dose, affecting subjective compliance and non-study co-interventions.
**Domain 3: Risk of bias due to missing outcome data**	Low Risk	Low Risk	Low and balanced attrition rate between study arms (<5%); reason for missing data accounted for and unlikely to bias results.
**Domain 4: Risk of bias in measurement of the outcome**	Low Risk	Some concerns	Overall survival is objective. PFS was centrally adjudicated by blinded independent radiologists (BICR), minimizing measurement bias. However, safety profile (AE attribution) and PROs are subjective and potentially vulnerable to

			detection/reporting bias due to open-label administration.
**Domain 5: Risk of bias in selection of the reported result**	Low Risk	Low Risk	Study conducted according to a pre-registered protocol (ClinicalTrials.gov) with pre-specified outcome measures and statistical analysis plan (SAP).
**Overall Risk of Bias**	**Low Risk**	**Some concerns**	**Objective efficacy outcomes carry low overall risk of bias; subjective outcomes carry 'some concerns' due to open-label design limitations.**

Table S2. Domain-level rationale and supporting judgments for the outcome-specific Cochrane Risk of Bias 2 (RoB 2) assessment of the RASolute 302 trial.
