# Supplementary material for: Efficacy and Safety of Daraxonrasib (RMC-6236) in Previously Treated Metastatic RAS-Mutated Pancreatic Ductal Adenocarcinoma: A Systematic Review
Source: Cancers (Basel). 2026 Sep 25;18(19):3112. doi: 10.3390/cancers18193112 (PMC13664388; doi:10.3390/cancers18193112)
Supplement: Supplementary file 1 [file cancers-18-03112-s001.zip › Table S3 .pdf]

| MINORS Item / Domain | Score (0–2) | Rationale and Supporting Judgments |
|---|---|---|
| **1. A clearly stated aim** | **2** | The study objective (evaluating safety, tolerability, PK/PD, and preliminary antitumor efficacy of RMC-6236 in RAS-mutated solid tumors/PDAC) was clearly defined. |
| **2. Inclusion of consecutive patients** | **2** | Consecutive eligible patients meeting defined inclusion/exclusion criteria were prospective enrolled across 16 clinical centers. |
| **3. Prospective collection of data** | **2** | All protocol-specified baseline parameters, safety data, and efficacy outcomes were collected prospectively according to a pre-established clinical protocol. |
| **4. Endpoints appropriate to the aim of the study** | **2** | Primary endpoint (safety/tolerability) and secondary endpoints (ORR, DCR, PFS, OS, DoR according to RECIST 1.1) were clearly defined and standard for Phase I/II oncology trials. |
| **5. Unbiased assessment of the study endpoint** | **1** | Open-label design without blinded central radiology review for early phase 1 expansion cohorts (RECIST 1.1 investigator assessment), introducing potential detection bias. |
| **6. Follow-up period appropriate to the aim of the study** | **2** | The median follow-up period was adequate to capture key efficacy endpoints (PFS, OS, DoR) and acute/long-term toxicities. |
| **7. Loss to follow-up less than 5%** | **2** | High patient retention was maintained; lost to follow-up was <5%, and all patient dispositions were fully accounted for in the ITT/evaluable populations. |
| **8. Prospective calculation of the study size** | **1** | Sample size for phase I dose escalation followed standard 3+3 design, and phase II cohort size was based on preliminary activity thresholds rather than formal prospective power calculations for subgroup comparisons. |
| **Total MINORS Score** | **14 / 16** | **High methodological quality (Low overall risk of bias)** |

**Table S3.** Methodological Index for Non-Randomized Studies (MINORS) assessment of the single-arm RMC-6236-001 trial.
